# Supplementary material for: Microarray-Based Detection of Antibodies against SARS-CoV-2 Proteins, Common Respiratory Viruses and Type I Interferons
Source: Viruses. 2021 Dec 20;13(12):2553. doi: 10.3390/v13122553 (PMC8705234; doi:10.3390/v13122553)
Supplement: Supplementary file 1 [file viruses-13-02553-s001.zip › Savvateeva et al, Figure S1.pdf]

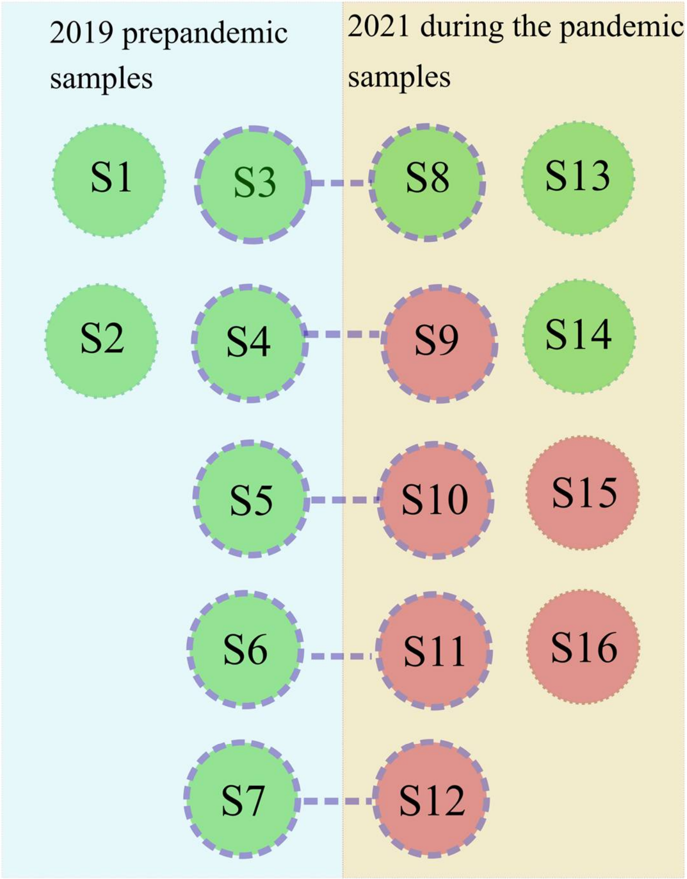


**Figure S1.** APS-1 patient's samples collected in 2019 and 2021. Green circles represent serum samples from APS-1 patients who have not been infected with SARS-CoV-2, and red circles show samples from COVID-19 recovered patients. Paired prepandemic and pandemic samples are highlighted with a dashed line.
